# Altered expression of testis-specific genes, piRNAs, and transposons in the silkworm ovary masculinized by a W chromosome mutation

**DOI:** 10.1186/1471-2164-13-119

**Published:** 2012-03-28

**Authors:** Kahori Hara, Tsuguru Fujii, Yutaka Suzuki, Sumio Sugano, Toru Shimada, Susumu Katsuma, Shinpei Kawaoka

**Affiliations:** 1 Department of Agricultural and Environmental Biology, Graduate School of Agricultural and Life Sciences, The University of Tokyo, Yayoi 1-1-1, Bunkyo-ku, Tokyo 113-8657, Japan; 2 Department of Medical Genome Sciences, Graduate School of Frontier Sciences, The University of Tokyo, 4-6-1 Shirokanedai, Minato-ku, Tokyo 108-8639, Japan

## Abstract

**Background:**

In the silkworm, *Bombyx mori*, femaleness is strongly controlled by the female-specific W chromosome. Originally, it was presumed that the W chromosome encodes female-determining gene(s), accordingly called *Fem*. However, to date, neither *Fem *nor any protein-coding gene has been identified from the W chromosome. Instead, the W chromosome is occupied with numerous transposon-related sequences. Interestingly, the silkworm W chromosome is a source of female-enriched PIWI-interacting RNAs (piRNAs). piRNAs are small RNAs of 23-30 nucleotides in length, which are required for controlling transposon activity in animal gonads. A recent study has identified a novel mutant silkworm line called KG, whose mutation in the W chromosome causes severe female masculinization. However, the molecular nature of KG line has not been well characterized yet.

**Results:**

Here we molecularly characterize the KG line. Genomic PCR analyses using currently available W chromosome-specific PCR markers indicated that no large deletion existed in the KG W chromosome. Genetic analyses demonstrated that sib-crosses within the KG line suppressed masculinization. Masculinization reactivated when crossing KG females with wild type males. Importantly, the KG ovaries exhibited a significantly abnormal transcriptome. First, the KG ovaries misexpressed testis-specific genes. Second, a set of female-enriched piRNAs was downregulated in the KG ovaries. Third, several transposons were overexpressed in the KG ovaries.

**Conclusions:**

Collectively, the mutation in the KG W chromosome causes broadly altered expression of testis-specific genes, piRNAs, and transposons. To our knowledge, this is the first study that describes a W chromosome mutant with such an intriguing phenotype.

## Background

In the silkworm, *Bombyx mori*, females are heterogametic (ZW) whereas males are homogametic (ZZ) [1,2]. Genetic studies have shown that at least one copy of the W chromosome is sufficient for determining femaleness, irrespective of Z chromosome copy number, suggesting that the W chromosome is a strong female-determinant in the silkworm [3,4]. Therefore, it was presumed that the W chromosome likely encodes a female-determining gene(s) so-called *Fem*. However, not even a single protein-coding gene has been identified from the W chromosome. Instead, it was found that the silkworm W chromosome contains numerous transposable elements, their remnants, and simple repeats [1,5-7]. These make it difficult to obtain a long contig sequence of the W chromosome, and thus the complete sequence of the W chromosome remains to be resolved.

Recently, we uncovered an intriguing facet of the silkworm W chromosome-the W chromosome is a source of female-enriched PIWI-interacting RNAs (piRNAs) [8]. PIWI proteins and PIWI-interacting RNAs are at the heart of transposon silencing system in animal gonads [9-11]. piRNAs are 23-30 nucleotide-long small RNAs that can act as sequence-specific guides for PIWI proteins. Mutations in piRNA pathway proteins lead to de-silencing of transposons and result in severe developmental defects in germ line cells [9-11]. With the aid of piRNA deep-sequencing and careful analyses, we identified a number of transposons and associated piRNAs originating from the sex-determining region of the W chromosome [8]. The role of female-enriched piRNAs remains enigmatic.

*Doublesex *(*dsx*) gene is an evolutionarily conserved transcription factor that mediates the somatic sex determination pathway [12,13]. *B. mori dsx *(*Bmdsx*) is alternatively spliced in a sex-dependent manner, yielding male-specific BmDSX or female-specific BmDSX (BmDSXM and BmDSXF, respectively). BmDSXF is a functional switch at the bottom of sex determination pathway in the silkworm [12,13]. *B. mori *P-element somatic inhibitor (BmPSI) and IGF-II mRNA binding protein (BmIMP) contribute to sex-specific splicing of *Bmdsx *mRNAs [14,15].

The KG line is a mutant line that shows various degrees of female masculinization features, such as formation of chitin-like structures in the external genitalia [16]. It has been genetically shown that masculinization is caused by a mutation in the KG W chromosome. One of the most prominent phenotypes in masculinized females is the aberrant splicing of *Bmdsx *mRNAs. Masculinized female tissues, including ovarian tissues, express both female- and male-type *Bmdsx *mRNAs, indicating that the mutation in the KG W chromosome interferes with the somatic sex determination pathway. Although this remarkable phenotype is unique to the silkworm KG line the molecular nature of KG W chromosome is largely unknown. Here, we investigated expression profiles of testis-specific genes, piRNAs, and transposons in the KG ovaries. Our results revealed that the mutation in the KG W chromosome caused misexpression of testis-specific genes, reduction of a set of piRNAs, and overexpression of transposons in the KG ovaries.

## Results and discussion

### Genetic analyses of the KG line

**Figure 1:**
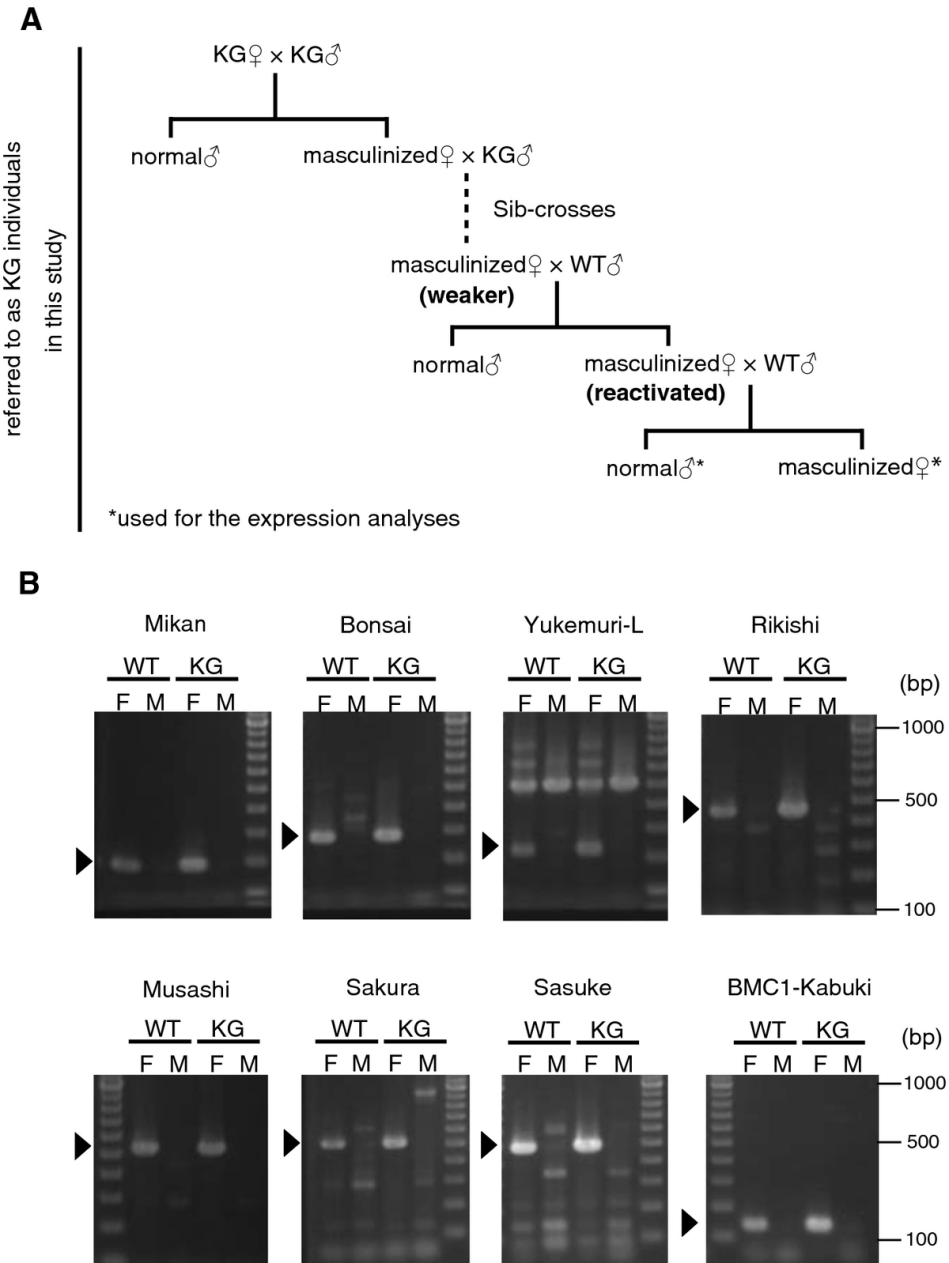
**Genetic analysis of the KG line**. (A) Mating scheme. KG♀×KG♂ and KG♀×WT♂, but not WT♀×KG♂ generated masculinized females. Sib-crosses weakend masculinized phenotypes, but KG♀×WT♂ reactivated masculinization. (KG♀×WT♂)♀×WT♂ individuals were used in this study. See also Table 1. (B) The molecular structure of KG W chromosome. Genomic PCR analyses were performed by using eight W chromosome-specific RAPD markers [17]. Arrowheads indicate W chromosome-specific amplicons.

The KG line had been maintained by continuous sib-crosses as is the case for other silkworm mutants. Unexpectedly, continuous sib-crosses between KG females and males over several generations weakened masculinized phenotypes (only 1/79 females showed masculinization at generation 4 (G4): Figure 1A and Table 1). Crossing a KG female of weakened masculinization with a WT male again produced severely masculinized females (187/187: Figure 1A and Table 1). Crosses using a WT female and a (G3♀ ×WT♂) male did not produce masculinized females (0/117: Table 1), confirming that the mutation resides in the KG W chromosome. These results indicated that masculinized phenotypes of the KG line were unstable to be genetically maintained.

**Table 1:** Genetic analysis of the KG line

Crosses	Female Masculinized	Female Normal	Male Normal
G0**♀ **× G0♂	0	1	2
G1♀ × G1♂	12	9	19
G2♀ × G2♂	35	92	140
G3♀ × G3♂	1	78	95
G3♀ × WT♂	187	0	201
WT♀ × (G3♀ × WT♂)♂	0	117	128

The numbers of masculinized females and males from four generations are indicated.

All G0 females exhibited masculinized phenotypes

Our results suggested that the mutation in the KG W chromosome became suppressed during sib-crosses. We envision that masculinized phenotypes depend on interaction between the mutated W chromosome and normal autosomes/Z chromosomes. For example, a masculinizing factor is located on autosomes/Z chromosomes while the W chromosome harbors an anti-masculinization factor, which might be mutated in the KG W chromosome. Thus, the results of sib-cross experiments can be explained by incompatibility between a masculinizing and anti-masculinization factors of the WT and the original KG line, respectively. Natural selection against the severe KG phenotypes may attribute to the diminishing masculinization as well.

Several W chromosome-specific RAPD makers are available for the silkworm W chromosome [17-19]. We utilized these markers to understand the structure of KG W chromosome. As shown in Figure 1B, when compared to the WT W chromosome, the KG W chromosome retained 8 RAPD markers surveyed, indicating that the KG W chromosome does not have a large deletion, unlike is observed in the W chromosome of sex-limited yellow-cocoon strain [8,17].

### KG ovaries misexpressed a number of testis-specific genes

To understand the molecular nature of the masculinized phenotypes observed in the KG line, we focussed on the masculinized ovaries. To obtain females showing severe masculinization, we used individuals generated by (KG × WT) female × WT male. For simplicity, these are described as the KG females/males or the KG line in this manuscript (Figure 1A).

**Figure 2:**
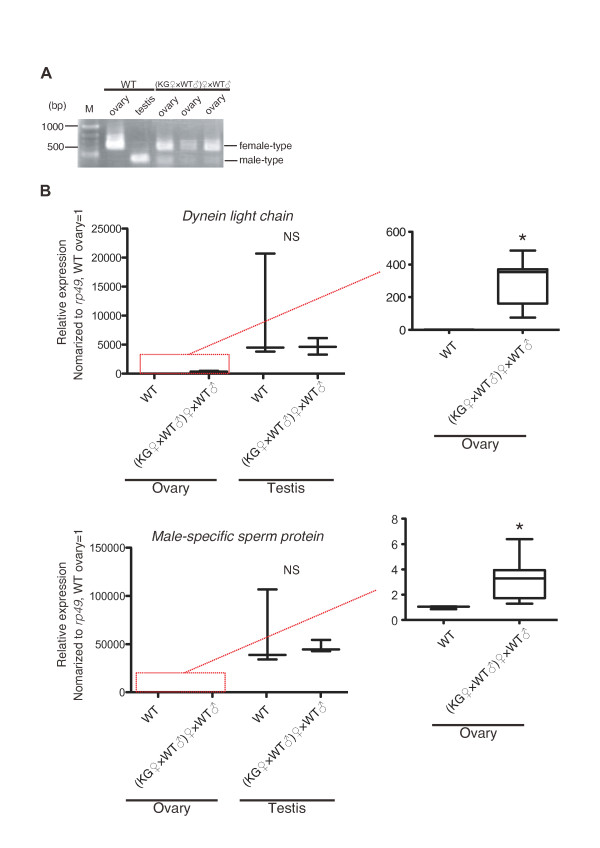
**Misexpression of testis-specific genes in the KG ovaries**. (A) Abnormal splicing of *Bmdsx *mRNAs in the KG ovaries. The primers that can discriminate female- and male-type *Bmdsx *were used. (B) Misexpression of testis-specific genes in the KG ovaries. The amounts of indicated mRNAs were measured by qPCR, normalized to *rp49*, and described as a box plot. Expressions of testis-specific genes in the WT and KG ovaries were enlarged. n = 3 for the WT ovaries, WT testes, and KG testes. n = 8 for the KG ovaries. * *p *< 0.05, Student's *t*-test. NS, non significant.

We at first investigated the splicing pattern of *Bmdsx *mRNAs. We confirmed that, as reported in Fujii et al. (2010) [16], the KG ovaries expressed both male- and female-type *Bmdsx *mRNAs, suggesting that the KG ovaries contained both female- and male-type cells (Figure 2A). At this point, we cannot exclude the possibility that a mutated single cell expressed both types of *Bmdsx *mRNAs.

To determine if the mutated KG W chromosome affects expression of testis-specific genes, we examined the abundance of several testis-specific mRNAs via quantitative PCR (qPCR). For this analysis, we focused on previously reported testis-specific genes [20]. Our results demonstrated that, when compared to the WT ovaries, several testis-specific genes were overexpressed in the KG ovaries (Figure 2B). The expression levels of these genes in the KG ovaries were still less abundant than those in the KG testes. The expression levels were quite similar between WT and KG testes. These data indicated that the mutation in the KG W chromosome disrupted sex-specific transcriptome in the KG ovaries. At this point, we cannot determine if misexpressions of these testis-specific genes are simply dependent on heterogeneous *Bmdsx *expression in the KG ovaries.

### Reduction of female-enriched piRNAs in the KG ovaries

We previously reported that the silkworm W chromosome is a source of female-enriched piRNAs [8]. With the aid of our guidelines, we can deduce genomic origins of transposons based on piRNA expression data-piRNAs derived from the W chromosome are expressed more abundantly in the ovary than in the testis. Thus, piRNA expression data from the KG ovaries are likely to be useful to understand the molecular nature of KG W chromosome. To this end, we constructed, sequenced, and analyzed piRNA libraries from KG ovaries of the three individuals generated by (KG × WT) female × WT male. Cloned reads were mapped to the silkworm genome to infer a sequencing depth for normalization. We focused on piRNAs mapped to 121 well-annotated transposons.

In the WT ovary, transposon piRNAs are enriched for U at the 5' ends (1U bias), and antisense piRNAs are more abundant than sense piRNAs (antisense strand bias) [8]. These biases were unaltered in the KG ovaries, suggesting that the mutation does not affect general properties of ovarian piRNAs (Table 2).

**Table 2:** 1U and antisense bias of piRNAs deriving from the KG ovaries

	WT	KG lines
1U bias (%)	73	69(± 0.91)
antisense bias	3.5	3.7(± 0.31)

The percentages of piRNAs with 1U in total transposon piRNAs are indicated

The ratios between sense and antisense transposon piRNAs are indicated

The standard deviations (n = 3) are shown in parentheses

**Figure 3:**
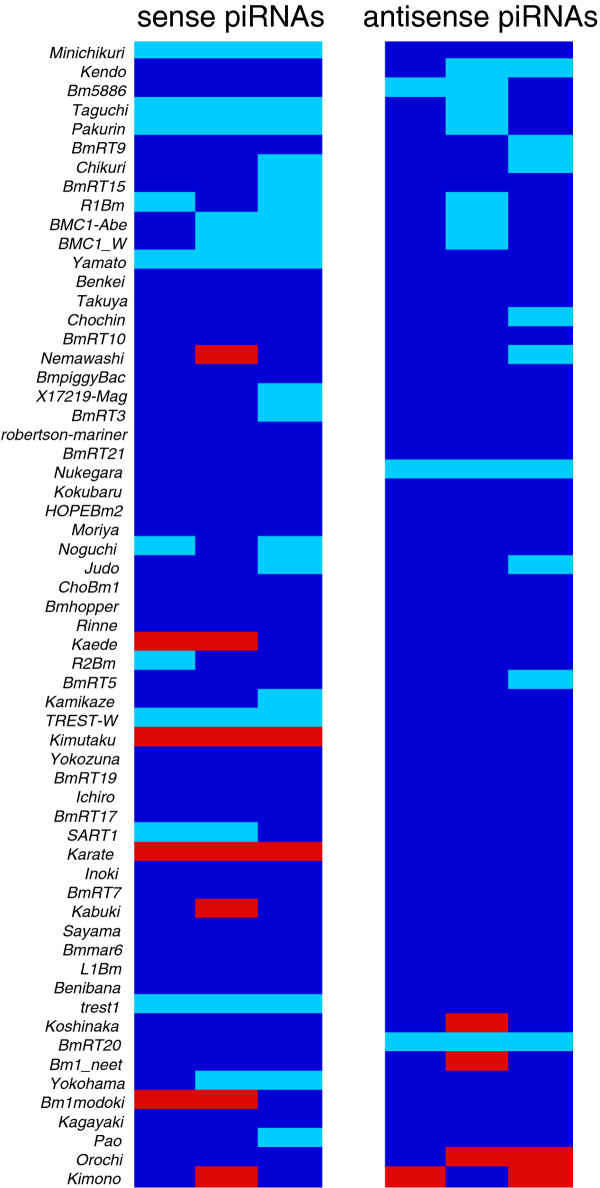
**Profiling of piRNAs in the KG ovaries**. Heat map showing relative abundances of sense and antisense transposon piRNAs in the KG ovaries of three individuals (WT ovarian piRNAs = 1). Blue indicates relative expression (RE) meets 0.6 ≤ RE ≤ 1.6. Light blue corresponds to RE < 0.6 and red indicates RE > 1.6.

**Figure 4:**
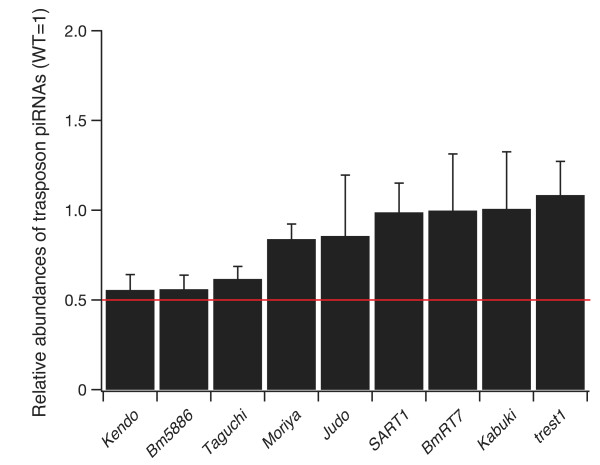
**Profiling of piRNAs deriving from the sex-determining region in the KG ovaries**. Relative abundances of total transposon piRNAs enriched in the sex-determining region [8]. Error bar indicates ± standard deviation (n = 3).

Next, we catalogued expression levels of sense and antisense transposon piRNAs. For this analysis, 60 transposons, to which more than 500 reads per million (RPM) ovarian piRNAs mapped, were used. We found that a set of sense and antisense transposon piRNAs including female-enriched piRNAs showed a significant reduction in the KG ovaries (Figure 3). When focused on female-enriched piRNAs deriving from the sex-determining region in the W chromosome [8], *Kendo*, *Bm5886*, and *Taguchi *piRNAs were significantly decreased in the KG ovaries (approximately 40% reduction: Figure 4). In contrast, although *trest1 *piRNAs are enriched in the sex-determining region [8], they were unaltered by the mutation in the KG W chromosome. Moreover, *Pakurin *female-enriched piRNAs, which are not enriched in the sex-determining region [8], exhibited a significant reduction in the KG ovaries (Figure 3). Taken altogether, the mutation in the KG W chromosome affected expression levels of a specific set of piRNAs including female-enriched piRNAs, but this was not restricted to piRNAs deriving from the sex-determining region. Disruption of female-enriched piRNA expressions could simply be attributed to an indirect consequence of male-like gene expression profiles established in the KG ovaries. Alternatively, considering that the mutation resides in the KG W chromosome, reduction of female-enriched piRNAs might be directly caused by the mutation in the KG W chromosome. Unfortunately, the unavailability of the entire W chromosome sequence and the lack of crossover in silkworm females make it impossible to determine the exact nature of mutation in the KG W chromosome.

### Overexpression of transposons in the KG ovaries

**Figure 5:**
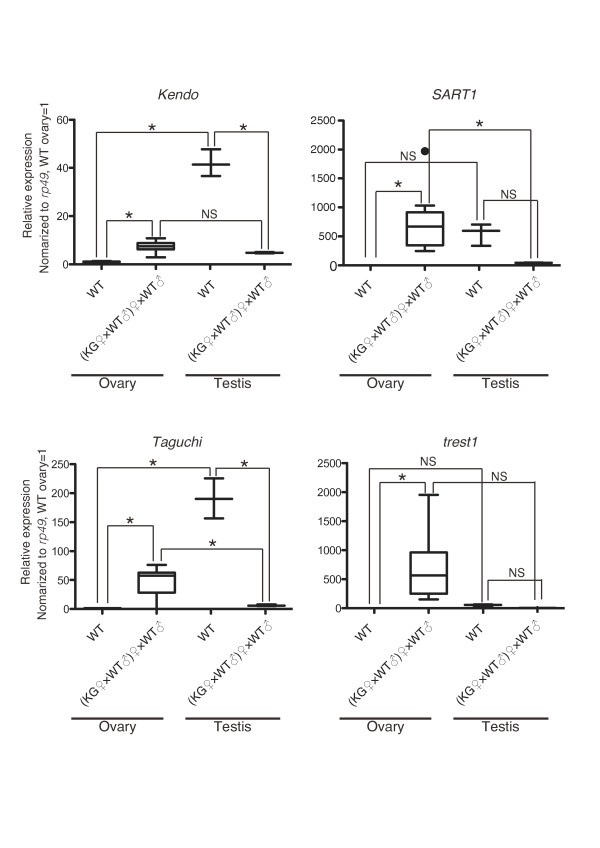
**Overexpression of transposons in the KG ovaries**. The amounts of indicated mRNAs were measured by qPCR, normalized to *rp49*, and described as a box plot. n = 3 for the WT testes and KG testes. n = 6 for the WT ovaries. n = 8 for the KG ovaries. * *p *< 0.05, one-way ANOVA, Tukey's multiple comparison test. NS, non significant.

Because we identified abnormal female-enriched piRNA expression, we set out to investigate the expression profile of several transposons (Figure 5). We detected higher expression of transposon RNAs in the KG ovaries than in the WT ovaries. Transposon RNAs were more highly expressed in the WT testes than in the WT ovaries. Expression levels were comparable (*Kendo *and *Taguchi*) or even higher (*SART1*, and *trest1*) in the KG ovaries than in the KG testes. These cases were different from the cases for testis-specific genes, whose expressions were higher in the WT and KG testes than in the KG ovaries (Figure 2B). Thus, it is likely that the observed overexpression of transposons could be caused by two independent phenomena: masculinization and transposon de-silencing. We suggest that the mutation in the KG W chromosome caused transposon de-silencing in the KG ovaries. Previous studies have demonstrated that piRNA reductions are not always coincident with corresponding transposon de-silencing [21-23]. Consistent with this, we observed piRNA reduction for *Kendo *but not for *SART1 *while both elements were overexpressed in the KG ovaries. Thus, it is still unclear whether reduction of female-enriched piRNAs is a direct cause for transposon de-silencing.

## Conclusion

Here we thoroughly analyzed an intriguing silkworm mutant called the KG line, whose mutated W chromosome causes severe female masculinization. Our genetic analyses showed that masculinization became suppressed during the course of sib-crosses over generations, but became reactivated when crossing a KG female with a WT male. These data indicated an interaction between the W chromosome and autosomes/Z chromosomes regarding masculinized phenotypes. Interestingly, the KG female ovaries misexpressed testis-specific genes. Expression levels of these testis-specific genes in the KG ovaries were higher than in the WT ovaries but less than in the WT testes. Comprehensive piRNA profiling revealed that a set of female-enriched piRNAs including those deriving from the sex-determining regions was reduced in the KG ovaries. Accordingly, we detected transposon de-silencing in the KG ovaries. Our current study suggests a possible link between the sex differentiation pathway and the piRNA pathway in the silkworm.

## Methods

### *Bombyx mori *strains

Wild type (WT) strain p50T (WT) and the KG line were reared on fresh mulberry leaves in an insect-rearing chamber under short-day conditions (12 L: 12D). Every batch was divided into two groups: one was for collecting ovaries and testes at the pupal stage and another was for confirming masculinized phenotypes at the adult stage.

### Genomic PCR

Genomic PCR with several W chromosome-specific RAPD markers was performed as described previously [17].

### piRNA library construction

Total RNA was prepared using Trizol reagent (Invitrogen, CA, USA) according to the manufacturer's protocol. The total RNA (10 μg) was loaded onto a 15% denaturing polyacrylamide gel containing 8 M urea, electrophoresed, and then stained with SYBRGold (Invitrogen). Signals were visualized using LAS-1000 film (Fujifilm, Tokyo, Japan). As silkworm piRNAs are visible as a distinct band by SYBRGold staining, we could easily gel-purify piRNA-containing fraction. Small RNA libraries were constructed using a small RNA cloning kit (Takara, Kyoto, Japan). DNA sequencing was performed using the Solexa genetic analysis system (Illumina, CA, USA). One nanogram of the prepared cDNA was used for the sequencing reactions with the Illumina GA. 10,000-15,000 clusters were generated per "tile", and 36 cycles of the sequencing reactions were performed. The protocols of the cluster generation and sequence reactions were according to the manufacturer's instructions.

### Sequence analysis

Solexa sequencing generated reads of up to 36 nucleotides in length. The 3' adaptor sequences were identified and removed, allowing for up to two mismatches. Reads without adaptor sequences were discarded. Reads shorter than 23 nucleotides or longer than 30 nucleotides were excluded, resulting in reads of 23-30 nucleotides. Alignment to the *B. mori *genome [24], 121 annotated transposons, and 1668 ReAS clones [25] were performed with SOAP2 (ver. 2.20) allowing no mismatch [26]. The total number of perfect genome-mapping reads reflects the sequencing depth. To compare the reads among different data sets, reads were expressed in reads per million (RPM) by normalizing to the total number of perfect genome-mapping in each library. Raw excel data for transposon piRNA profiles in the KG ovaries will be provided upon request.

### Quantitative PCR (qPCR)

cDNA synthesis and quantitative PCR analyses were performed as described previously [27]. Primers used in this experiment are described in Table 3.

**Table 3:** Primers used in this study

Name	Seq. (5' to 3')
*Male specific sperm protein*-RT-Fw	GCGAGAGGTGCTTATGGTTC
*Male specific sperm protein*-RT-Re	GTCCATGGTGCGCATAAATA
*Dynein-light-chain*-RT-Fw	GGTTTGGGCAAAAAGACAGA
*Dynein-light-chain*-RT-Re	ATCTTTTGGTTTCGCCAGTG
*Kendo*-RT-Fw	CAAACGTGCGCGCAAGTCCG
*Kendo*-RT-Re	ATACGGTCGTCGTGCCGGAG
*SART1*-RT-Fw	TCGCAGGCAGTTATTGTCAA
*SART1*-RT-Re	GGCTAAAATCGATACGGCAGA
*Taguchi*-RT-Fw	CCCTTTGCACTGACAAAGAG
*Taguchi*-RT-Re	TGTGGGCGATGGACAGGCTT
*trest1*-RT-Fw	TTGCGTCCACCGCTTCCTCC
*trest1*-RT-Re	TGTGTTGAGACATTCCGCGG
*Bmdsx*-Fw	AACCATGCCACCACTGATACCAAC
*Bmdsx*-Re	GCACAACGAATACTGCTGCAATCG

### Data deposition

piRNAs sequenced in this study are deposited as DRA000275 in the DNA database of Japan (DDBJ).

## Abbreviations

piRNA: PIWI-interacting RNA; RAPD: randomly amplified polymorphic DNA.

## Competing interests

The authors declare that they have no competing interests.

## Authors' contributions

HK performed most molecular experiments and participated in manuscript preparation. TF and TS performed genetic analyses. YS and SS performed solexa-sequencing analysis. SuK supervised the study and participated in manuscript preparation. ShK analyzed deep sequencing data, supervised the study, and wrote the paper. All authors read and approved the final manuscript.
